# Patients' perspectives on a patient-oriented electronic decision support tool to reduce overuse of proton pump inhibitors (arriba-PPI): a qualitative study in primary care

**DOI:** 10.1186/s12875-023-01991-0

**Published:** 2023-01-25

**Authors:** Alexandra Schmidt, Bettina Bücker, Michaela Maas, Susanne Löscher, Annette Becker, Annika Viniol, Julia Heisig, Stefan Wilm, Anne Barzel

**Affiliations:** 1 Chair of General Practice II and Patient-Centredness in Primary Care, Institute of General Practice and Primary Care (IAMAG), Faculty of Health, Witten/Herdecke University, Witten, Germany; 2 Institute of General Practice, Medical Faculty, Heinrich-Heine-University Duesseldorf, Duesseldorf, Germany; 3 Chair of General Practice I and Interprofessional Care, Institute of General Practice and Primary Care (IAMAG), Faculty of Health, Witten/Herdecke University, Witten, Germany; 4 Institute of General Practice, University of Marburg, Marburg, Germany; 5 Department of General Practice and Primary Care, Ulm University Hospital, Ulm, Germany

**Keywords:** Arriba-PPI, Proton pump inhibitors, Shared decision-making, Qualitative interviews, General practice, Primary care, Germany

## Abstract

**Background:**

To evaluate patients’ perspectives and their experiences with a consultation involving a computer-assisted and patient-centered discontinuation strategy (arriba-PPI tool) as part of a German multicenter study on reducing the prescription of proton pump inhibitors (PPIs).

**Methods:**

Qualitative in-depth telephone interviews on proton pump inhibitors with patients who had received an arriba-PPI tool-based counseling by their general practitioner (GP). A random sample of 30 patients was taken from study participants. Interviews were conducted in 2020 and analyzed using a thematic qualitative text analysis.

**Results:**

Although this was meant to be the key to shared decision making with regard to PPI reduction, study participants mostly did not recall the visual features of the tool. However, a few patients remembered them very clearly. Above all, patients appreciated a trustful relationship with the GP as well as comprehensive, individualized counseling.

**Conclusion:**

Application of the arriba-PPI tool can support the decision process but can also hinder the consultation process if the tool is not properly embedded in the consultation. GPs using the arriba-PPI tool to support the shared decision-making process should consider the patients’ and their own expectations on the benefit of the visual representation of the tool.

**Supplementary Information:**

The online version contains supplementary material available at 10.1186/s12875-023-01991-0.

## Background

Proton pump inhibitors (PPIs) are drugs that effectively suppress gastric acid secretion [1]. Accordingly, PPIs are used for the treatment of peptic ulcers, reflux esophagitis, non-specific chronic gastritis, and Barrett's esophagus. PPIs have significantly improved the treatment and prophylaxis of the above-mentioned diseases [2–6], and are thus on the WHO Model List of Essential Medicines [7]. Over the past decade, PPI usage has increased considerably. In Germany, PPI prescriptions in 2018 were more than twice as high as ten years ago [8].

However, recent studies have shown increasing evidence of serious side effects of long-term PPI therapy, such as osteoporotic fractures, pneumonia, and renal insufficiency [9–12]. Considering these new findings on serious side effects the steady increase in PPI prescriptions needs to be reevaluated, particularly with regard to the proper indication [2, 13–15]. To address this problem we developed and subsequently tested a novel computer-based and patient-centered tool (arriba-PPI). The electronic decision support tool arriba consists of several modules that have been scientifically evaluated previously. The arriba-PPI tool is targeted at the primary care setting to support GPs to identify and reduce inappropriate long-term prescribing of PPIs and was developed in a feasibility study before. Arriba-PPI was developed with the aim to support a joint decision for or against discontinuing PPI using a visual feature (traffic light display) and a printout for recommendations on discontinuation to check on current indication for the consulted patient. The traffic light display helps to clarify whether stopping PPIs is recommended or not. Green indicates the clear recommendation for a withdrawal, yellow indicates that withdrawal usually is recommended, and red indicates that withdrawal usually is not recommended. For more details see the RCT study protocol [2]. Main quantitative results from the RCT will be published soon. We also conducted interviews with the GPs involved in the study, the results will be published individually.

Embedded in this study on the effectiveness of the tool, we conducted a qualitative sub-study to gain insights into the practical application of the arriba-PPI tool and to take a closer look at the consulting experiences on PPIs in general and on potentially occurring difficulties in reduction and discontinuation attempts. The results can help to evaluate the arriba-PPI tool from the patients’ perspectives and to provide recommendations for a future implementation of the arriba-PPI tool in routine care. Therefore, in this sub-study we looked at the patients’ point of view to answer the following two research questions:

- 1. How did patients experience counselling with the arriba-PPI tool?
- 2. What factors influence attempts to reduce and/or stop taking PPIs?

To evaluate the research questions in context, topics such as patients’ medical history, experience with and evaluation of different treatments, knowledge and feelings about the illness, relationship und communication with GP, lifestyle, and the extent of personal control over symptoms were addressed additionally.

## Methods

We opted for an exploratory study design based on semi-structured interviews to gain an in-depth understanding of patients’ experiences with the arriba-PPI tool. This study is reported according to the COREQ guidelines [16].

### Participants and setting

Semi-structured interviews were considered the most suitable method to investigate participants’ ability to comprehend information and to explore personal opinions on consultations with the arriba-PPI tool. We conducted in-depth telephone interviews with patients belonging to the intervention group of the arriba-PPI study. Written consent was obtained from all study participants. Patients were recruited by the study centers Witten/Herdecke University, Duesseldorf University and Marburg University and came from cities and rural areas of the Ruhr and Rhein valley in North Rhine-Westphalia and the area of North and Middle Hesse, Germany. In total, the arriba-PPI study included 2,387 patients, 1,256 of whom received a consultation with the arriba-PPI tool by their GP (intervention group). Patients with a regular prescription of PPIs of ≥ 6 months were included. We defined regular prescription as taking at least one PPI pill daily or as taking regularly several PPI pills per week (such as four pills per week/every other day). Potential interview partners were randomly selected from an intervention group sample of patients (*n* = 461) who had previously agreed to be contacted for a telephone interview before the telephone interviews started in January 2020.

### Data collection and analysis

A semi-structured interview guide was developed using the method: Collect, Review, Sort, Summarize [17]. Within interdisciplinary group meetings involving all co-authors the following topics were consented: history of PPI intake, attempts of reduction and stopping PPI, experiences with doctor-patient communication, and finally experiences with the arriba-PPI tool: “[….] Now please think back to your doctor’s appointment [date], where your intake of [medication] was discussed. Please tell me what you remember and how you felt about this appointment.”

We followed the information power concept [18] and carried out interviews until we had sufficient information power for the analysis of our research questions as well as for the generated quality of the dialogue. Considering the main question with regard to the arriba-PPI tool, we began interviews with patients who had previously clearly stated to remember the arriba-PPI-tool seeing on screen during the consultation at the practice (*n* = 12). We also interviewed patients, who were not sure whether they had seen the tool on screen (*n* = 18), to evaluate the impact of consultation with the tool without showing the visual contents or the consultation itself (intention-to-treat analysis).

A research associate of the arriba-PPI project with a master in Social Sciences (AS) was trained and carried out the interviews. The interviewer did not know the participants or their GPs. At the beginning of each interview, it was made clear that the interviewer was independent and that opinions in all directions could be expressed openly without any consequences. Reasons for an interest in the research topic were stated by the interviewer.

For analysis, all interviews were taken with anonymized verbatim transcripts of full length. To systematically organize data into a structured format a deductive-inductive approach based on thematic qualitative text analysis according to Kuckartz [19] was used. The transcripts were coded independently by two researchers (AS and MM) to increase the reliability of the study. In the first step of the coding process, the entire material was coded using the main categories, extracted from the interview guide. Additionally the transcripts were partially coded with two further researchers (SW, BB) to improve the initial coding system. All passages were assigned to the main categories, and sub-categories were determined inductively from the material. In a second step, all data were coded according to the finally evolved coding system yielding a category-based analysis along the main topics. Any differences with regard to understanding of topics or assignment to categories were solved by discussion. The coding process resulted in five main topics (consultation appointment with arriba-PPI tool, doctor-patient communication, health problems/symptoms, PPI intake, and intake of stomach remedies other than PPI), and 25 sub-categories. An excerpt of the coding scheme is supplied in an additional file (Additional file 1)*.* The software MAXQDA 20 was used for analysis and coding process. Interviews were analyzed in German. The analysis was continuously discussed and refined in a multiprofessional group (social science, practice assistant, GPs, public health).

## Results

### Participant characteristics

Thirty interviews were conducted between January 2020 and June 2020, including 14 men and 16 women. The age of the interviewees ranged from 45 to 82 years (mean 65.4 years, SD ± 9.9 years, IQR 10 years). With regard to average age and sex distribution, the characteristics were similar to the arriba-PPI study, with 54.4% female participants and an age ranging from 18 to 95 years (mean 64.5 years, SD ± 12,9 years, IQR 19 years). The time range between the arriba-PPI tool consultation and the interview varied from 4 to 14 months (mean 8 months). The duration of the interviews ranged between a minimum of 6:23 min and a maximum of 41:39 min (mean 13:29 min). Of the 38 patients contacted for an interview request, six patients refused participation and two patients were scheduled for an interview but could not be reached by phone. Also, the duration of PPI medication varied considerably between the participating patients, ranging from less than 1 year to longer than 20 years. The mean intake duration of PPI medication in the main study was 5.3 years (SD ± 4.39).

### How did patients experience the counselling with the arriba-PPI tool?

Considering patients experience on doctors’ consultation using the arriba-PPI tool, the main category “consultation appointment with arriba-PPI tool” was used to evaluate the encounter of the study patients with the novel arriba-PPI tool.

Apparently, the arriba-PPI tool was not used in all GP-PPI medication counseling appointments. According to the interviews, only 17 out of 30 interviewees reported some kind of counselling with the arriba-PPI tool during their GP visit. Some interviewees remembered the visual tool contents spontaneously or on request, or the printout they received at the end of their consultation on PPI medication. But, in some patients, no evidence of counseling with the tool was apparent, and for other patients it remained unclear whether the tool was used for counseling. Overall, eight patients who recalled the visual content remembered the tool either positively or negatively. Only two patients remembered the traffic light display of the arriba-PPI tool in a clearly positive way.

“[…] and it actually did help me in some way, the traffic light, absolutely. I take on a more conscientious approach now, you know? I no longer just swallow a pill when I have problems with my stomach, sometimes I just drink chamomile tea instead.” (female, 69)

“But, in my head and just the thought that I could get down to yellow, the yellow traffic light, that made me happy, of course.” (male, 65)

One patient, however, associated the tool with rather negative memories:

“A doctor always radiates more than just his knowledge. A doctor always radiates competence and empathy and it all works together, kind of like a counseling package. […] And for me […] when someone reads questions from the computer screen, […] and you recognize that it is not the doctor, but that he is just reading it from somewhere […] then it did not reach me that the questions were simply being read off and I recognized straight away what direction we were heading towards.” (male, 52)

In some cases the tool did not seem to meet the aim of supporting the GP in the consultation. The quoted statement indicates that contents of the tool may appear redundant. It was also mentioned that the GP acts as a "counseling package", highlighting the function of the counselor him-/herself. The GP’s communication skills appear to be of key importance.

In summary, it can be stated that despite the sample of interviewees (*n* = 30), which mostly included patients who explicitly remembered a consultation with the tool (*n* = 17), the (visual) tool itself was clearly remembered only by a few patients. With regard to the appointment, interviewees spontaneously remembered participating in the study rather than the counseling appointment with the arriba-PPI tool. It was also positively noted that participation in the study led to a discussion on the use and necessity of PPI medication by the GP. One potential explanation could be the time span with an average of 8 months between the appointment on PPI medication.

### What factors influence attempts to reduce and/or stop taking PPIs?

With regard to potential influencing factors on taking PPIs, the main categories doctor-patient communication, health problems/symptoms, PPI intake and stomach remedies other than PPI were taken into account. Apparently, in this group of 17 interviewed patients consultation with the tool did not necessarily lead to a modified PPI intake. For 12 patients no change in their PPI dose was documented, four patients successfully reduced their PPI dosage, and one patient successfully stopped taking PPI:

“So for me it was good, […] that one doesn’t always have to take pills, pills, and more pills. Instead, I actually dealt with the whole situation, and if I don’t really have to take pantoprazole, well then I don’t take it. In this regard I trust Dr. R. and I think that that is fine.” (female, 71)

Since this patient was almost blind, she did not look at the visual arriba-PPI features during the consultation. Nevertheless, the stepwise reduction of her PPI intake after the counseling was successful. This case highlights some effective factors for successful stopping and reduction attempts: a trustful relationship with the GP and feeling well cared for during the consultation seem to be the most important influencing factors for a change in attitude towards taking PPIs. With regard to the GP consultations, many patients felt well informed and cared for, although some expressed a lack of consultation und communication. When the reduction attempts were initiated at the GP’s request (as a result of study participation), the PPI intake remained the same after a short period of time, as exemplified by the following quote:

„And he said it is possible that I don’t really need it, the omeprazole, and because of that I should absolutely try to stop taking it, but in small steps, he said. But that didn’t work, so I just started taking 20 mg of omeprazole again.” (male, 64)

This patient reported that his GP said he should "absolutely" try to stop taking PPIs. But the patient "just” continued to take his medication again as before. It seemed there was a lack of internal motivation for this patient.

Further, some patients stated that they could not live without their PPIs and mentioned a fear of pain or a past experience of discomfort when reducing or stopping PPIs, such as problems sleeping without the medication or feeling that their stomach would not function properly without it. Statements like the following demonstrate the potential for being dependent on the drug:

“But I don’t know whether 20 mg every second day may be enough. I don’t want to try that though, I’m too afraid to try. […] afraid that I would get severe pain again which is so EASY to prevent by taking medicine.” (male, 52)

“And when I notice that there are only four or five tablets left for the last / for the next week. Then it’s time / then I quickly order the next pack (both laugh). You’re not trying to take that away from me, are you?” (female, 66)

In summary, we can establish that, for patients, PPI medication seems to be of reasonable importance with regard to the expected quality of life and well-being. Moreover, patients may refuse to reduce or stop their PPI intake because of the proven symptom relief or anticipation thereof. Apparently, the decision on PPI reduction depends on factors during the consultation itself. If a consultation led to/addressed the patients’ personal desire to reduce PPIs, there was a chance that reduction or even termination of the PPI medication could be achieved. Thus, the arriba-PPI tool can support decision making in a conversation between the GP and the patient, but it is not recognized/perceived as absolutely indispensable.

## Discussion

In this study, we evaluated patients’ perspectives and experiences with a consultation using a computer-assisted and patient-centered discontinuation strategy (arriba-PPI tool) to take a closer look at important factors influencing consultations on PPI intake. The results of our analysis indicate that the arriba-PPI tool itself did not primarily lead to a shared decision-making process. However, according to a systematic review computerized decision aids have been shown to improve at least one patient-centered outcome, and the quality of the digital aids used was particularly important [20]. Given the fact that many patients in our study had been taking PPI for several years, it is questionable whether the arriba-PPI tool was able to provide additional information on PPI medication. Moreover, we found that successful reduction and discontinuation attempts still depended on individual and trust-based counseling and that especially fears regarding a life without PPI play a major role for PPI patients. A scoping review underlines these results; successful reduction of PPI intake appears to depend on trustful and comprehensive counseling [15].

Our findings are in line with previous studies on PPI which reported that patients perceive benefits from taking PPIs besides the management of symptoms, such as improved quality of life, which is why they are scared of reducing or discontinuing their PPI intake [14, 21]. Qualitative research on GPs’ perspective shows that GPs believe that patients could manage their symptoms without PPI if they modify their lifestyle accordingly; but instead, they prescribe PPIs as an effective way of reducing their workload in terms of repeated consultations and examinations [14]. Moreover, if the drug is taken over a long period of time, there also may be a temporary increase in acid secretion after stopping PPI. This can lead to recurrence of symptoms regardless of the status of the underlying disease [2, 8] and can increase patients’ inhibitions towards reducing or stopping the medication, especially if previous attempts have failed [15]. As a result, the process of stopping PPIs is more time-consuming and difficult than continuing to prescribe the drug, which is why GPs perhaps avoid sometimes investing the necessary time in consultations [14, 22]. The results on GPs’ and patients’ perspectives highlight the challenges faced in consultations regarding PPI reduction.

## Limitations

One limitation of the study is the time frame of 4 to 14 months (mean 8 months) between the doctors’ counselling on PPI embedding the arriba-PPI tool, and the interviews carried out for this sub-study, potentially causing distorted or faded memories. Conducting interviews on the telephone may present a limitation compared to conducting them face-to-face. Non-verbal and personal communication is limited. Further, the study participants’ interest in information on reduction and side effects of PPI may have been limited due to their predominantly long time intake of PPI. Furthermore, the experience of an in depth talk with their GP about health problems and PPI intake as described by some patients may have been induced more by the patient's GP participating in the study and less initiated by the arriba-PPI tool itself.

## Conclusion

While regular counseling on the use of PPIs is particularly important in long-term users, there seems to be a lack of counseling. Our research shows that counseling on PPIs can be successful in terms of reducing unnecessary PPI intake either with or without the consultation tool. It is important to explicitly address patients' concerns and fears and involve them in shared decision-making on further use, reduction, or discontinuation. The computerized decision tool arriba-PPI is apparently not necessarily needed, but can support GPs in this process, especially if the tool application is part of the counseling process. Psychological and social factors, such as the relationship with the treating GP, have a more significant influence on patient-centered counseling outcomes. A trustful relationship can help reduce fears about managing symptoms without PPI that are associated with reducing and stopping attempts. This also suggests that the implementation of the tool in the doctor-patient conversation should strengthen the trustful relationship with the patient and ideally lead to a shared decision. Therefore, GPs should assess individually whether the patient could benefit from the visual features, or whether use of a computer-based consultation would hinder a joint decision-making process.

This research was undertaken to explore the experience of PPI patients with a novel computer-based and patient-centered tool (arriba-PPI). By describing the consultation appointment on PPI medication in the context of everyday life we were able to detect the role of the arriba-PPI tool in counseling on PPI medication and other important factors on stopping/reducing PPI intake. This allowed us to draw a conclusion on the practical application of the tool in PPI consultations and to make recommendations for consultations on PPI medication in general. From the patients point of view the tool was rarely a key point in consultation. However, they may benefit from the tool as it is reminding GPs to evaluate continued use of PPI with long-term users.

## Supplementary Information

**Additional file 1. **Example of citations for the individual categories of thedeductive-inductive category system (patients).

## Data Availability

The data analysed within study is available from the corresponding author on reasonable request.

## Abbreviations

PPI: Proton Pump Inhibitor
arriba-PPI: Novel patient-centered tool for shared decision making on PPI use
GP: General practitioner
