# Supplementary material for: Patients' perspectives on a patient-oriented electronic decision support tool to reduce overuse of proton pump inhibitors (arriba-PPI): a qualitative study in primary care
Source: BMC Prim Care. 2023 Jan 25;24:33. doi: 10.1186/s12875-023-01991-0 (PMC9875449; doi:10.1186/s12875-023-01991-0)
Supplement: Supplementary file 1 — Additional file 1. Example of citations for the individual categories of thedeductive-inductive category system (patients). [file 12875_2023_1991_MOESM1_ESM.docx]

**Additional file 1: Example of citations for the individual categories of the deductive-inductive category system (patients)**

| **Main category** | **Subcategory/s** | **Examples of citations** |
| --- | --- | --- |
| **Consultation appointment with arriba-PPI tool** | Feedback on the study in general | “I don’t remember. All I know is that afterwards, he / it took forever until he even heard about it. And then I said that the whole study, well that it was going down the drain anyway and the fact that somebody should contact me after such a long time, well, I did think that was a little odd. You always get harassed on the phone and give / normally I don’t talk about these things on the phone, you know?” (212221_w_1951, Pos. 46) |
|  | Recollection of consultation within the study | “Yes, for me that was a completely normal doctor’s visit. We spoke about the pantoprazole, how well I tolerate it and whether it helps me etcetera. I don’t remember the details anymore.“ (114619_m_1972, Pos. 30) |
|  | Recollections of the screen contents | "I: Can you still remember that? Could you tell me what you remember about THAT particular appointment? Cw: Uhm, well I was there quite often. And I remember that afterwards I sat at the computer with Mrs. S. and she also did this traffic light system with me, I think. There was also an additional program that we discussed; I think it was also from you somehow." (212912_w_1974, Pos. 27-28)  "No, we didn’t have a screen." (211106_m_1965, Pos. 64) |
|  | Printout (from arriba-PPI tool) | "A printout? No, I didn’t get one." (1140017_m_1968, Pos. 46) |
|  | Changes after consultation | "I: That means there were changes after the appointment / Tw: There were none, no. No changes." (114607_w_1949, Pos. 27-28)  "Well, he did say that I should see to it that I lower the dose, you know? That I should lower the dose, always further down. I remember that I was taking 40 after the surgery, you know? I don’t know, was it four or even six weeks? Then he asked, make sure you stick to the time so that we can continue to lower the dose. Then we always / then 20, yes. Only. And now I am down to 10, that’s what I use now. I halve them, with 10, when I need to. Just so that I can reduce the dose / there’s always the fear. You simply can’t do that from one day to the next. You know? The body has just gotten so used to it, always, with everything, you know? Yes, yes." (312219_w_1938, Pos. 56) |
| **Doctor-Patient Communication** | Communication with GPs | "Nobody EVER asked me about it or anything. I was just always happy, when I / not happy. Just like always, she wrote me a prescription and that was it. So she / I don’t know either." (311606_w_1954, Pos. 72) "Then we tried taking that for a few weeks, the feeling of something obstructing my throat never really got better, okay, and then we reduced it again, and now we are down to 20 mg." (215905_m_1975, Pos. 12) |
|  | Communication with other physicians | "I then decided to first speak to the cardiologist who I have to visit for regular checkups anyway and he said to me: "Yes, well you know, one doesn‘t always notice a stomach bleed right away and that is why it is important to take preventive action ". Etcetera, and because that can happen more commonly with the drug, with ASA / okay. Then I took it for another six months." (114607_w_1949, Pos. 6) |
|  | Opinions about doctors in general | "Yes, like always. You know, they don’t really have much time anyway, you know. The doctors, their offices are always overcrowded. And who do you see there? It’s usually the young people, you know? Young people, most of them are young, just like this morning." (312219_w_1938, Pos. 58)  "A doctor always radiates more than his knowledge. A doctor always radiates competence and empathy and it all works together, kind of like a consultation package, where you sometimes already feel better just because you have spoken to a doctor." (114605_m_1968, Pos. 38) |
| **Health Problems/**  **Symptoms** | Medical examination procedures | "I don’t really remember a lot because I have been taking it for so long. But I do remember that I had heartburn quite often at the time, and also some problems with my stomach, and then once they just carried out a gastroscopy." (215905_m_1975, Pos. 6)  "Yes. Yes. And then they / I stopped taking pantoprazole and I was feeling very poorly. I had VERY BAD stomach problems then. And then I had a gastroscopy and colonoscopy." (212221_w_1951, Pos. 22) |
|  | Diagnoses | "Then I went to see the doctor once and he carried out a gastroscopy, he said everything was alright and then he sent me home again." (311606_w_1954, Pos. 8)  "I don’t really have any problems with that, my stomach, concerning my stomach I am relatively robust. I have absolutely no problems with my stomach, but apparently taking Rabo / pantoprazole was necessary because of the other drugs I was taking, to make sure it is all well tolerated." (315113_m_1939, Pos. 8) |
|  | No or hardly any problems | "You take one of those per day, in the morning, and, well, I obviously didn’t notice any difference because I never really had any problems with my stomach before that. And then I, so, I started taking the medication on / from the beginning, after they discharged me and / well let me think about it. About two years ago I asked myself: "Well, actually my stomach feels good / it always felt good. So why should I take this stuff?" (114607_w_1949, Pos. 6) |
|  | First indication/first problems/reason for using PPI | "So then / I had / I didn’t really have pain in my stomach, the doctor prescribed it as a precaution. Because of all these/ I also had cancer and at the time I was still taking cancer medicines and yes." (114811_w_1949, Pos. 16)  "I only ever took the ome / omeprazole. Because I always had acid that came / it kept creeping up and then I couldn’t burp. That’s how I would describe it. It was excruciating." (214402_w_1943, Pos. 6)  "It was only about that; I always had a bad case of heartburn." (211205_m_1945, Pos. 4) |
|  | Conditions that lead to taking PPIs (in progress) | "[…] and if I then take ibuprofen or something similar at higher doses because of some kind of pain, I sometimes end up getting heartburn." (215905_m_1975, Pos. 8)  "Precisely. Basically the nausea always stayed the same, but since I started taking 20 mg, the nausea has gotten worse again" (211119_w_1950, Pos. 24) |
|  | Conditions that improve the problems | “So, I stopped smoking, that is a very relevant factor / that’s right, I forgot about that. I stopped smoking in 2000.“ (114605_m_1968, Pos. 10)  "So, in the phases where the dose was continuously lowered to 20 mg every second [day]. Those phases of my life were quite stable. Things were actually going well at the time. Everything was normal. And there were no changes, neither in a positive nor in a negative sense, but everything was very, very good. Things were going well." (114605_m_1968, Pos. 22) |
|  | Conditions that worsen the problems | "It depends on what I eat. If it’s too salty or so, then / or too fatty, but actually I avoid that now, eating fatty foods. But when it’s too salty, then I also get heartburn. Or if I eat foods that are sweet, or fruit, mango or so, then I also get heartburn. Tomatoes, really anything that is acidy. I should actually avoid those foods, but when I feel like them, I just eat them. And then I suffer. (Laughs)" (311617_w_1957, Pos. 8) |
|  | No conscious changes in life circumstances | "Actually, in this regard, nothing has changed. The only thing I ever really got when I took that medicine was a slightly / pretty light stool. Not like usual, like normal, but it had a pretty light color." (114609_m_1949, Pos. 8) |
|  | Rebound effect on discontinuation/reduction | "Actually I have more or less been taking it regularly ever since. Once we did stop the medication for a while, but then I got heartburn again and then we started taking it straight away again. And then it actually got a lot better again." (215905_m_1975, Pos. 6)  "Much better. I didn’t really notice anything anymore, I didn’t have any stomach pain, nothing really. I have been taking it for many years now and I never had any more problems." (310210_w_1956, Pos. 22) |
| **PPI intake** | Intake schedule | "I always try to take it around / two hours before going to bed, so usually at around 8 a.m. and at 6 p.m., so I take it at 8 p.m. and at 6 p.m. is when I eat my last meal. After that I drink only water." (1140017_m_1968, Pos. 14) |
|  | No changes in the dosage | "I: Had you changed your dose again since then? Did you stop taking the medicine or lower the dose? CCM: No, that’s always the way it has been, yes." (315113_m_1939, Pos. 15-16) |
|  | Dosage increase unrelated to the study | "The initial idea was clear then, come on, let’s put up the dose, let’s increase it, so then we doubled the dose. So currently I am taking 20 mg once daily and then we changed to / no, we started with 40 mg once daily, [...]" (215905_m_1975, Pos. 12) |
|  | Dosage reduction unrelated to the study | "Yes, one year ago my doctor lowered the dose to 20 mg, just to try it out, to see if that would be enough for me and if not, she said she would change it again, change it back to 40 mg. Now I have tried that for a year, but it hasn’t really gotten any better, nothing really, and so, basically / Yes, the nausea has gotten worse, that much I can say. And one week ago I started taking 40 mg again." (211119_w_1950, Pos. 21) |
|  | Perception of PPIs | "That’s a long time, that’s how long I have been taking this pantoprazole. And the only thing I noticed with this pantoprazole is that I my stools are quite light, which wasn’t the case before." (114609_m_1949, Pos. 60)  "Yes, well the / the doctors are negative. They have such different opinions and that is really negative and it’s also supposed to be very harmful for the body, isn’t it? That is why I am a little scared in that regard, that is why I only take it spora / so only when I need it." (212221_w_1951, Pos. 38) |
|  | Total duration of intake | "And then in 1997, I think, they put me on pantoprazole to begin with, I think that’s what it was, and then later they gave me omeprazole." (114605_m_1968, Pos. 6)  "That was ten years ago. And they prescribed it to me that because I had a stomach ulcer […]" (114820_w_1947, Pos. 6)  "Yes, I believe I took it for five or six years." (310210_w_1956, Pos. 6)  "Oh, boy, that is a long time ago, that was a few years ago. I can’t tell you with absolute certainty, you see." (312219_w_1938, Pos. 8) |
| **Intake of Stomach remedies other than PPI** | Taking other stomach remedies | "Yes, I had already tried that before as well. Iberogast, then this / I don’t know the names of them all, the ones you can get from the pharmacy, the over-the-counter medicines. But none of them really helped very much." (114619_m_1972, Pos. 20) |
|  | No other stomach remedies | "No, I did not take anything. I just decided to change the supplier this time. Before I was using a different one, and now I got the same medication from a different manufacturer, I have that now, but I didn’t notice a difference. That is the only one I took though, that is correct.“ (1140017_m_1968, Pos. 22) |
